# Infectious Disease Sensitivity to Climate and Other Driver‐Pressure Changes: Research Effort and Gaps for Lyme Disease and Cryptosporidiosis

**DOI:** 10.1029/2022GH000760

**Published:** 2023-06-09

**Authors:** Y. Ma, Z. Kalantari, G. Destouni

**Affiliations:** ^1^ Department of Physical Geography Stockholm University Stockholm Sweden; ^2^ Department of Sustainable Development Environmental Science and Engineering (SEED) KTH Royal Institute of Technology Stockholm Sweden

**Keywords:** infectious disease, climate‐sensitivity, climate change, water, land, socioeconomics, transmission pathways, disease geography, Lyme disease, cryptosporidiosis, scoping review, research effort, research gaps

## Abstract

Climate sensitivity of infectious diseases is discussed in many studies. A quantitative basis for distinguishing and predicting the disease impacts of climate and other environmental and anthropogenic driver‐pressure changes, however, is often lacking. To assess research effort and identify possible key gaps that can guide further research, we here apply a scoping review approach to two widespread infectious diseases: Lyme disease (LD) as a vector‐borne and cryptosporidiosis as a water‐borne disease. Based on the emerging publication data, we further structure and quantitatively assess the driver‐pressure foci and interlinkages considered in the published research so far. This shows important research gaps for the roles of rarely investigated water‐related and socioeconomic factors for LD, and land‐related factors for cryptosporidiosis. For both diseases, the interactions of host and parasite communities with climate and other driver‐pressure factors are understudied, as are also important world regions relative to the disease geographies; in particular, Asia and Africa emerge as main geographic gaps for LD and cryptosporidiosis research, respectively. The scoping approach developed and gaps identified in this study should be useful for further assessment and guidance of research on infectious disease sensitivity to climate and other environmental and anthropogenic changes around the world.

## Key Points

- Studies on Lyme disease (LD) and cryptosporidiosis relationships with climate lack coupling with other environmental and anthropogenic factors
- Driver‐pressure interactions with host and parasite communities are generally understudied
- Asia and Africa are main geographic research gaps for LD and cryptosporidiosis research, respectively

## 1 Introduction

Impacts of climate change on infectious diseases may threaten human life or take a substantial economic toll on impacted societies (Limaye et al., 2019). Climate change influences transmission of infectious diseases through various direct and indirect ways. For example, if temperature rises above a certain threshold, increases are expected in mosquito biting rates and parasite replication within mosquitoes, along with mosquito development and mortality effects (Rohr et al., 2011), which in combination determine disease outbreaks in human communities. That is, climatic factors influence transmission pathways through effects on abundance of vectors like mosquitoes and ticks (Rogers & Randolph, 2006), pathogen survival outside the host(s) (Lowen et al., 2007), and overall community ecology and biodiversity that in turn also affect host‐pathogen interactions (Altizer et al., 2013; Callaghan et al., 2004; Pecl et al., 2017). Climate change can also affect exposure risk through its interactions with other environmental factors, such as water conditions in the landscape for water‐borne pollution and associated infections, for example, of cholera (Reiner et al., 2012) and by mobilization of previously frozen pathogens through permafrost thaw (Selroos et al., 2019).

Disease impacts may thus be amplified or counteracted by co‐occurring changes in climate and other environmental and anthropogenic drivers and pressures. The driver‐pressure terminology relates to the widely used “Driver‐Pressure‐State‐Impact‐Response” (DPSIR) framework of relevant causality indicators for environmental change and sustainable development (e.g., Malmir et al., 2021; Potschin, 2009) and these terms have also been used with analogous meanings in direct relation to climate change and infectious diseases (McMichael & Woodruff, 2008). The distinction between drivers and pressures depends to some degree on the specific application context of the DPSIR framework, which is why we here use the terms jointly as drivers‐pressures. Often, drivers in this framework represent socioeconomic changes and developments (e.g., in population or other demographic aspects, or in economic, agricultural or energy policies) (Malmir et al., 2021) but overarching climate change can also be considered a driver depending on specific framework application (Potschin, 2009). Pressures can further be natural or result from direct or indirect human interference, such as natural land‐cover changes in response to natural climate variability and change, or human land and water use changes in response to driving policy changes and/or anthropogenic climate change. Together, such driver‐pressure combinations can also lead to changes in disease occurrence.

For example, land‐use changes can alter vector, host and pathogen niches, and host and vector community composition, to posing either higher or lower infection risks (Bellard et al., 2012; Gottdenker et al., 2014; Ogden & Tsao, 2009; Randolph & Dobson, 2012). Moreover, landscape hydrological conditions are related to variations and change trends in both weather‐climate and human land‐ and water‐uses locally regionally (Destouni & Prieto, 2018; Jarsjö et al., 2012; Moshir Panahi et al., 2022) and globally around the world (Destouni et al., 2013; Jaramillo & Destouni, 2014; Kåresdotter et al., 2022) and their changes, for example, in flood event occurrence, can affect vector breeding sites and related vector‐borne disease outbreaks, as well as human exposure to water‐borne diseases (S. Y. Liang & Messenger, 2018). In some regions, disease impacts of landscape‐hydrology changes (Ma et al., 2021) or human activity developments (Reiter, 2001) can even outweigh those of climate change for various diseases (e.g., tick‐borne encephalitis, Q fever and Puumala virus infection for the former types of impacts, and malaria, yellow fever, dengue fever for the latter). In addition, such environmental and anthropological factors in turn also have feedbacks to climate change (Chahine, 1992; Destouni et al., 2010; Henderson‐Sellers, 1994; Jarsjö et al., 2012; Swingland et al., 2002; Williams et al., 2007), thus representing another indirect driver‐pressure pathway to possible disease impact. In general, a multitude of climatic and non‐climatic driver‐pressure factors and their interactions thus combine in determining the occurrence geographies and timings of various infectious diseases, as discussed in many theoretical, experimental and empirical studies (Ma et al., 2021; Moreira et al., 2020; Omazic et al., 2019; Semenza & Paz, 2021; Shuman, 2010).

To decipher the sensitivity of infectious diseases to combined changes in various climate, environmental and societal factors, and solve related health and societal problems, research needs to include multidisciplinary collaborations and systems thinking (McNutt, 2017). Due to the problem complexity and common limitations of available data and models, however, studies often focus on just some selected factor(s) and interaction(s). Such focus choices may limit and bias our understanding and predictive capability of disease sensitivity to climate and other driver‐pressure changes (Altizer et al., 2013; L. Liang & Gong, 2017). To assess these research choices and identify possible remaining key gaps and biases, we here develop and apply a scoping review type of approach (Desai & Zhang, 2021). Based on the publication data emerging from this scoping review, we further structure, explore and quantitatively assess the foci and factor interlinkages considered in published research so far on disease occurrence and sensitivity related to climate and other environmental and anthropogenic change drivers and pressures.

The scoping review is applied to two infectious disease examples, indicated in previous study as sensitive to changes in climatic and other environmental conditions, primarily related to temperature, precipitation and/or water flow variables (Ma et al., 2021): Lyme disease (LD) (borreliosis), a vector‐borne disease; and cryptosporidiosis, a water‐borne disease. Lyme disease is caused by the *Borrelia* burgdorferi sensu lato species complex, and is transmitted by vector Ixodidae ticks (Stanek et al., 2012). The main hosts for LD are divided into transmission hosts and reproduction hosts. Transmission hosts are infected small mammals, such as mice, voles and some species of birds that transfer pathogens to ticks (Ostfeld & Keesing, 2000). Reproduction hosts are animals, such as deer and cattle that facilitate long‐term survival of ticks but not themselves infected with LD (Gray et al., 1992, 1995; Jaenson & Tälleklint, 1992; Stanek et al., 2012). Cryptosporidiosis is caused by the cryptosporidium parasite, which infects the gastrointestinal epithelium, causing diarrhea that may even be life‐threatening in immunocompromised persons (Chen et al., 2002). The parasite can be transmitted from an infected host to a susceptible host by the faecal‐oral route (Fayer et al., 2000), and is resistant to commonly used disinfectants, which is a main public health concern (Peeters et al., 1989). Oocysts can persist in the environment (Robertson et al., 1992) and be readily mobilized by certain precipitation events (Davies et al., 2004).

These two diseases are selected for this scoping review as examples with a broad geographic distribution (Putignani & Menichella, 2010; Schotthoefer & Frost, 2015) and increasing trends in many regions (Gharpure et al., 2019; Hofhuis et al., 2015), but differing in their transmission pathways and hydro‐climatic sensitivities (Ma et al., 2021). There are further no vaccines available to the public for these diseases (Kamp et al., 2020; Silva et al., 2021) with potential severe consequences (Lindgren et al., 2012) that relevant research needs to provide support for societies to prevent and manage. As such, it is important to investigate and reveal the complexity aspects and driver‐pressure factors and impacts considered and linked in research so far, and identify possible key gaps and biases that need to be addressed in further research for each of these disease examples.

In particular, we address in this scoping review the following six main research questions for each investigated disease:

1. How has research effort evolved over time regarding disease sensitivity to climate and other driver‐pressure changes?
2. How many studies are quantitative, that is, provide a basis for mechanistic or statistical quantification of disease relationships with climate and other relevant environmental and anthropogenic changes, or for projection of future disease evolution scenarios?

and further for the quantitative studies (identified in answering question 2):

3 What study and quantification methods do they use?
4 What climatic and other co‐occurring environmental or anthropogenic driver‐pressure change factors do they investigate?
5 Which transmission pathway components are studied and to what degree are the components connected in the studies?
6 Which world regions are studied and how do they relate to the geographic disease distribution?

Based on the answers to these questions, we identify gaps of so far lacking but needed research for improving our understanding of infectious disease sensitivity to changes in climate and other environmental and anthropogenic drivers and pressures.

## 2 Materials and Methods

The literature searches for this scoping review were performed in Web of Science™ (WoS) and considered publications from 1 January 2000 to 10 February 2022. WoS is a commonly used database for scoping reviews (Dennen et al., 2020; Rokaya et al., 2018; Vigouroux & Destouni, 2022). It is also a multidisciplinary database, able to capture studies on climate and other environmental and societal change effects on infectious diseases across a broad range of research areas. Importantly, WoS can be expected to span a representative large sample of relevant papers for capturing the real distribution of relative shares of papers among various related topics, even if the absolute numbers of papers do not include all possible papers on each topic. Search terms were ((“borreliosis” OR “Lyme disease”) AND (“climate” OR “climate change” OR “climate variability”)) for LD, ((“cryptosporidiosis” OR “cryptosporidium” OR “crypto.”) AND (“climate” OR “climate change” OR “climate variability”)) for cryptosporidiosis. The search yielded 555 publication results for LD and 185 for cryptosporidiosis; the reference data and information extracted from these publications are listed and provided in an open‐access database (Ma et al., 2022). Preliminary screening was performed on these hits based on titles and abstracts to identify and exclude articles that: (a) were not about the targeted disease; (b) were conference abstracts; (c) did not consider both climate and disease; (d) for LD, were not about the *Ixodes* species transmission of the *Borrelia* pathogen; (e) were not written in English; and (f) were not full‐text open access.

**Figure 1:**
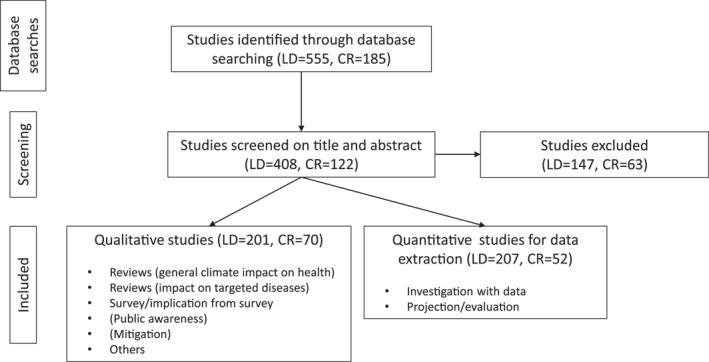
Work flow chart for the search of literature published from 1 January 2000 to 10 February 2022 and the distinction between quantitative and qualitative (descriptive) studies (LD: Lyme disease; CR: cryptosporidiosis). Parenthesized sub‐categories of public awareness and mitigation studies only apply to studies on Lyme disease, not to studies on cryptosporidiosis.

With regard to point (d), the *Ixodes* species in the final publication results include *Ixodes ricinus* (the main vector of Lyme borrelia in Europe), *Ixodes persulcatus* (the main vector in Asia), *Ixodes scapularis* (the main vector in northeastern and upper midwestern USA), *Ixodes pacificus* (the vector in western USA), and *Ixodes hexagonus* (the vector in Europe) (Gray, 1998), as well as *Ixodes angustus* based on clinical evidence that it is capable of occasionally transmitting *Borrelia burgdorferi* to humans (Damrow et al., 1989). Beyond these, not all *Borrelia* species are pathogenic. In North America, the only species known to cause LD in humans is *Borrelia burgdorferi sensu stricto*, while at least five species of Lyme borrelia (*Borrelia afzelii*, *Borrelia garinii*, *B*. *burgdorferi*, *Borrelia spielmanii*, and *Borrelia bavariensis*) may cause the disease in Europe (Margos et al., 2011; Nadelman & Wormser, 1998; Stanek et al., 2012). Among the studies addressing ticks or hosts, not all differentiate the genospecies of *Borrelia* species and we here include all those studies as long as they are about LD occurrence and risks related to climate. This approach resulted in a final total of 408 relevant studies for LD and 122 for cryptosporidiosis (Figure 1). To answer the first research question, on how research effort on climate and other driver‐pressure sensitivity of the two diseases has evolved over time, we assessed the distribution of publication times for this total number of relevant studies.

To address the second research question, we further distinguished between studies that are quantitative (as defined in the Introduction for question 2) and those that are qualitative (descriptive reviews, surveys, discussions on, e.g., possible climate impacts, social awareness, mitigation options; see also bullet‐list in Figure 1). Based on this distinction, the quantitative studies were found to be 207 (51% of total) for LD and 52 (43%) for cryptosporidiosis (Figure 1). To answer the remaining questions 3–6, these quantitative studies were further structured based on content, to identify and quantify research effort in terms of: (a) types of study methods (question 3); (b) studied categories of climate and other abiotic environmental and anthropogenic factors and types of variables within each category (question 4); (c) transmission pathway components and links between these addressed in the studies (question 5); and (d) regions studied around the world (question 6). This information was extracted from the methods section of each study or from the full text if necessary.

For question 3, the study methods were classified into four categories: laboratory/field experimentation/observation, statistical analysis, mechanistic modeling, or synthesis/meta‐analysis. A study may apply more than one method, thus repeat counting may occur. For question 4, we structured the climate and other factors considered in the different studies in five main categories of climatic factors and other environmental (land, water, other) and anthropogenic (socioeconomics, other) factors as outlined in Table 1.

**Table 1:** Categories of Factors and Variables Within Each Factor Category Studied in the Quantitative Publications

Category	Lyme disease		Cryptosporidiosis	
	Variable	Sub‐variable	Variable	Sub‐variable
Climate	Temperature		Temperature	
	Precipitation		Precipitation	
	Air humidity		Air humidity	
	Wind speed		Wind speed	
	Solar radiation		Solar radiation	
	Cloud cover		Extreme weather	Heavy rainfall, storms, extreme heat
	Climate variability	The North Atlantic Oscillation (NAO)	Climate variability	The Indian Ocean Dipole (IOD), The El Niño‐Southern Oscillation (ENSO)
			Air pressure	
Land factors	Ground properties	Temperature, soil features, litter depth	Ground properties	Soil features
	Vegetation	Density, height, vegetation type, forest type, vegetation water content, the Normalized Difference Vegetation Index (NDVI), beech tree production, pine masting, growing stage	Vegetation	Vegetation type, the Normalized Difference Vegetation Index (NDVI)
	Land use	Urban, agriculture, green cover, grazing, landscape fragmentation	Land use	Urban area, agriculture area
Terrestrial water factors	Surface water	Distance to coast, proximity to water source, river length, water‐covered area	Stream flow and quality aspects	Turbidity, flow rate, aquifer type, pH, electrical conductivity, water level
	Snow cover		Snow cover	
	Soil moisture		Soil moisture	
	Extreme hydrological events	Long‐term drought index	Extreme hydrological events	Long droughts, flood frequency, flood extent (area), flood history (event)
	Evapotranspiration	Potential evapotranspiration, actual evapotranspiration	Water resource facilities	Water supply, water treatment
Socioeconomics	Demography		Demography	
	Human awareness		Urbanization	
	Human behavior		Human behavior	
	Socioeconomic development		Socioeconomic development	
Other	Disease seasonality		Disease seasonality	
	Location‐related features	Latitude, longitude, altitude, day length, slope	Location‐related features	Latitude, longitude, altitude
	Wildfires			
	Biodiversity			

**Figure 2:**
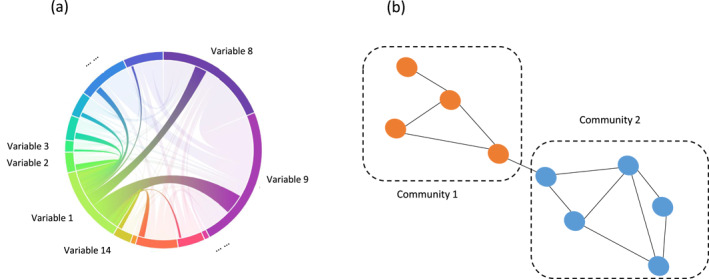
(a) Chord diagram representing paired interactions between different factors/variables (Table 1). (b) Network diagram showing in the same color factors/variables identified as belonging to the same community.

To quantify and illustrate studied interactions between different factors and variables in Table 1, we created chord and network diagrams, as shown schematically in Figure 2. A chord diagram (Figure 2a) shows paired factor/variable interactions, where the perimeter length associated with a factor/variable node represents (is proportional to) the number of studies that connect that node with other factor/variable nodes, relative to the total number of all studied node connections. The chord diagrams were created using the HoloViews Python library. The network diagrams (Figure 2b) were created using the Louvain algorithm for detection of connected factor/variable communities; the algorithm detects this by optimizing the network graph modularity, a measure of edge density within relative to outside of a factor/variable community (Blondel et al., 2008). By use of this algorithm, input factor categories and variables within them from Table 1 (represented by nodes in Figure 2b) are classified into different connected factor/variable communities. These communities show which factors/variables are mostly studied as connected (with connections represented by edges between nodes in Figure 2b) so that the algorithm characterizes them as falling within the same factor/variable community (nodes with same color in Figure 2b). The network diagram thereby reveals which factor interactions are mostly considered in the reviewed studies.

For question 5, we investigated which of transmission pathway components of the host‐vector‐human chain for LD and host‐pathogen‐human chain for cryptosporidiosis are considered and to what degree they are connected in the studies. For question 6, finally, we considered specific countries as the smallest scale for spatial resolution in investigating the geographic distribution of studied regions; smaller than whole‐country study sites were counted as studies of the associated countries. Studies of larger, transboundary regions around the world were considered separately.

## 3 Results

### 3.1 Research Effort and Its Evolution Over Time

**Figure 3:**
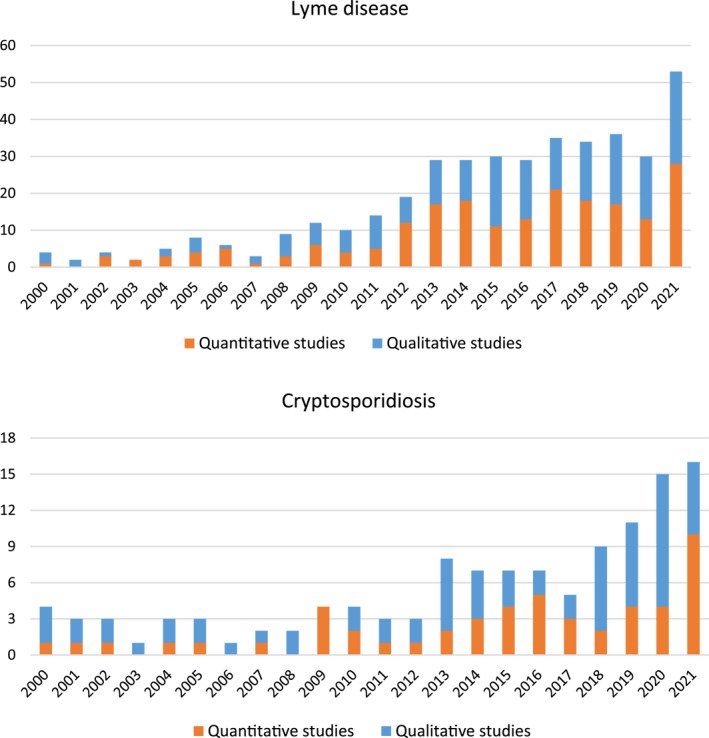
Total number of publications on (upper panel) Lyme disease and (lower panel) cryptosporidiosis over the period 2000–2021 (starting from 1 January 2000 and not showing in this illustration the small fraction of papers published in 2022, until 10 February, which were also included in the review). Orange and blue colors distinguish between quantitative and qualitative studies (as defined in Figure 1 and the Section 2).

Overall, research effort so far has been considerably greater for LD (408 in total) than cryptosporidiosis (122 in total), but both show a rapidly increasing trend in the last decade (Figure 3). This applies for the quantitative (orange), qualitative (blue) and total studies.

**Figure 4:**
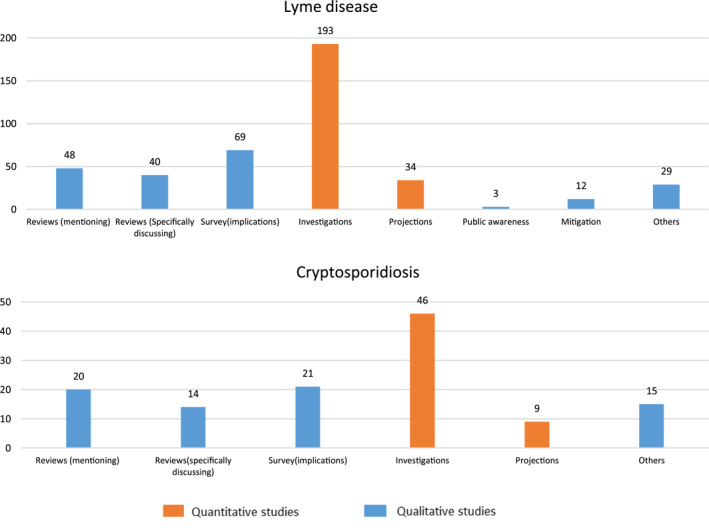
Counts of different sub‐categories of qualitative and quantitative studies for (upper panel) Lyme disease and (lower panel) cryptosporidiosis.

Figure 4 further shows the research effort distribution among different types of studies. The quantitative studies (orange) include mostly investigations of present and/or previous conditions (in short referred to as “investigations”; Figure 4 shows that these are: 193% or 47% of the total studies for LD; and 46% or 37% for cryptosporidiosis), and to much lesser degree projections/evaluations of future scenario conditions (34 for LD, 9 for cryptosporidiosis). The qualitative studies include: reviews mentioning or specifically discussing possible connections between climate and the two diseases (in total, 88 for LD, 34 for cryptosporidiosis); surveys that mention or discuss potential climate impacts (69 for LD, 21 for cryptosporidiosis); and various disease mitigation, public awareness and other discussion papers that also consider possible climate links (in total, 44 for LD, 15 for cryptosporidiosis).

### 3.2 Quantitative Study Methods

**Figure 5:**
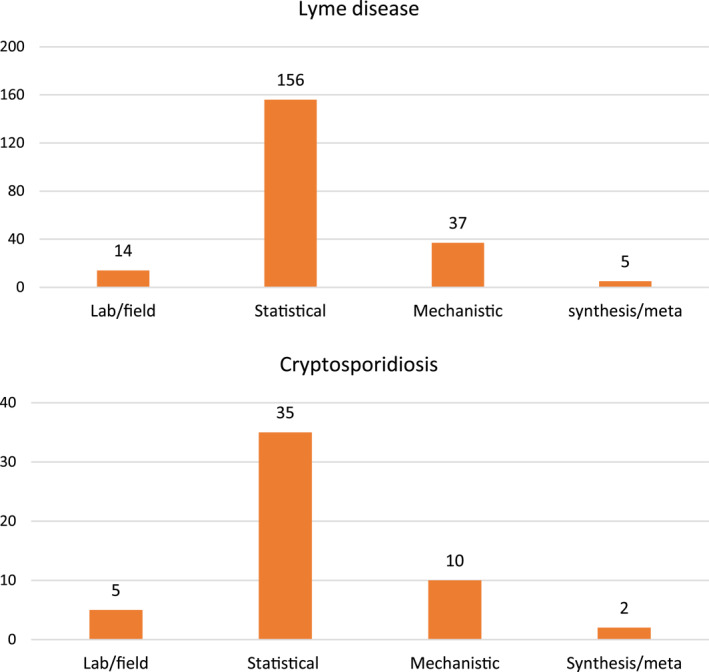
Methods used in the quantitative studies for (upper panel) Lyme disease and (lower panel) cryptosporidiosis.

The most common method used in the quantitative studies is statistical analysis (156 such studies for LD, 35 for cryptosporidiosis; Figure 5). To lesser degree, common methods further include mechanistic modeling (37 for LD, 10 for cryptosporidiosis), laboratory/field experimentation/observation (14 for LD, 5 for cryptosporidiosis), and/or synthesis/meta‐analysis (5 for LD, 2 for cryptosporidiosis) (Figure 5).

Statistical methods are likely used because their relative simplicity is advantageous, so that the correlations can be easily caught with even limited available data. However, they are also criticized for not quantifying actual infectious disease processes, that is, the specific interactions between the pathogenic microorganism, the climatic environment and the host. As such, statistical analyses, in particular of relatively small data samples, may mislead conclusions about causal relationships. In contrast, mechanistic modeling that aims to capture specific cause‐effect relationships in disease development and transmission (Cheng et al., 2016) can compensate for the drawbacks of statistical methods. For example, mechanistic models developed for LD have incorporated hosts, vectors, and their interactions, and can thereby project influences of temperature changes on population dynamics of ticks in various life stages (Wu et al., 2015), as well as quantify host community impacts on LD transmission (Lou et al., 2014; Ratti et al., 2021). Mechanistic modeling, however, also has drawbacks, such as requirements of large amounts of observation data with a certain resolution for application to specific locations, for which such data often do not exist (Lou et al., 2014; Ogden et al., 2005, 2013; Ratti et al., 2021).

### 3.3 Climate and Other Environmental and Anthropogenic Factors

**Figure 6:**
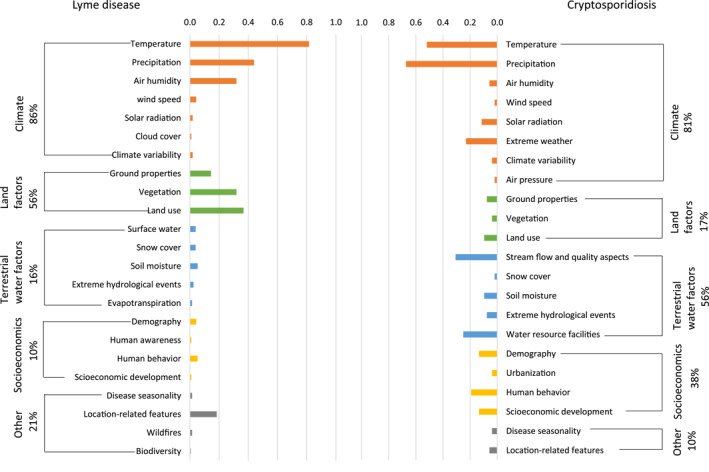
Main factors investigated in the published quantitative studies on (left) Lyme disease and (right) cryptosporidiosis. Variables are marked with different colors to differentiate the main categories they belong to. Percentages given are all in relation to the total number of quantitative studies.

Figure 6 summarizes the distribution of quantitative study effort among climate and other environmental and anthropogenic driver‐pressure factors (following the factor and variable structure in Table 1). In the climate factor category, temperature and precipitation are the most frequently investigated variables. For LD, the three most frequently investigated climate variables are temperature (82% of quantitative studies), precipitation (44%), and air humidity (32%). For cryptosporidiosis, they are precipitation (67%), temperature (52%), and extreme weather (23%). For LD, besides the climate factors (86%), 56% of studies consider also land factors, 16% of the studies consider terrestrial water factors and 10% consider socioeconomic factors. For cryptosporidiosis, besides the climate factors (81%), 56% of studies consider terrestrial water factors, 38% consider socioeconomic factors, and 17% consider land factors. Water and socioeconomic factors are thus relatively understudied for LD while land factors are understudied for cryptosporidiosis, even though these factors are also relevant for these diseases as discussed further below (see Section 4).

**Figure 7:**
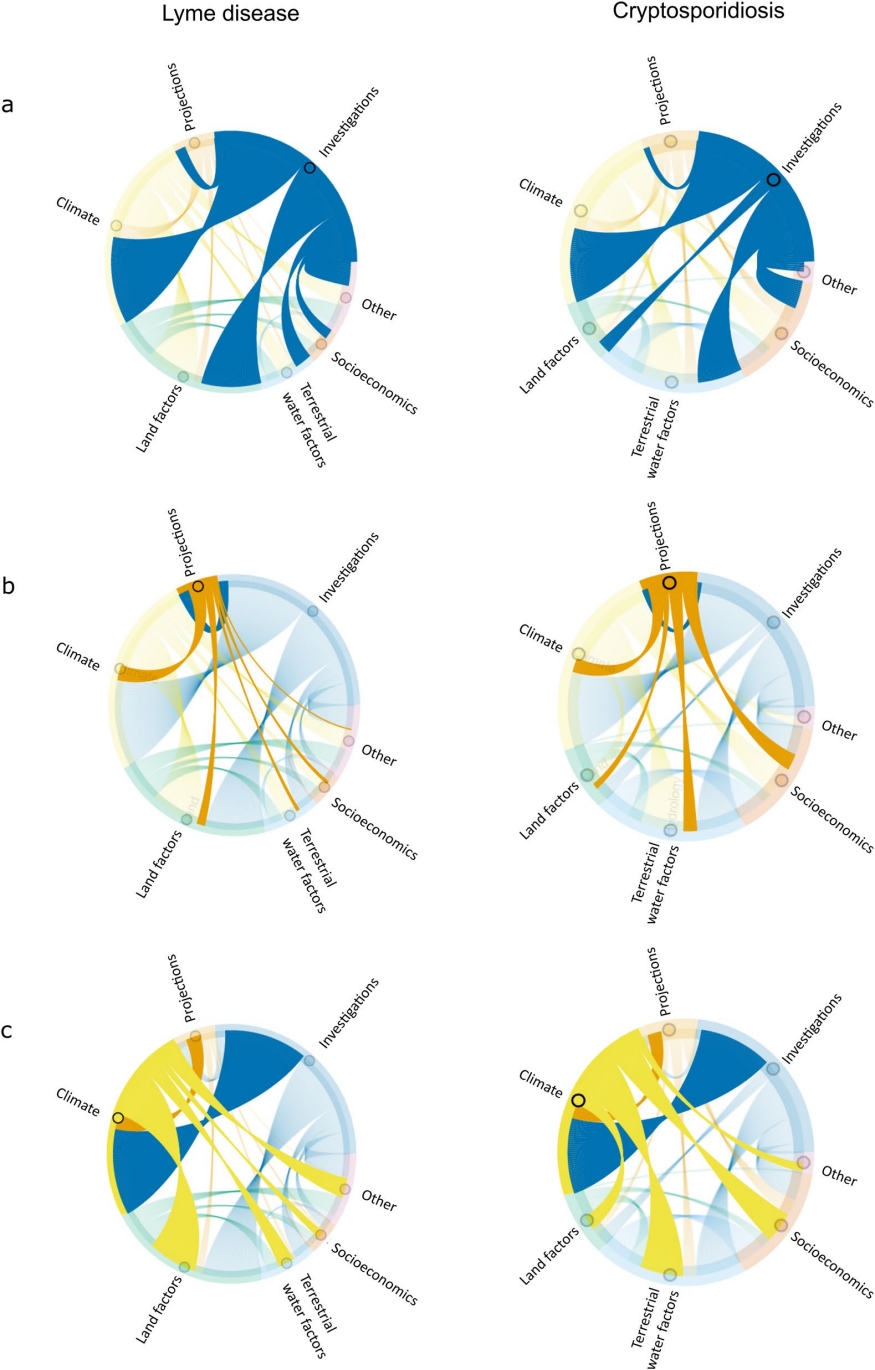
Chord diagrams emphasizing the different factors considered in: (a) investigation studies and (b) future projection studies; and (c) the paired connections between climate and other abiotic factors.

Chord diagrams for the categories of factors involved in investigation (Figure 7a) and projection (Figure 7b) studies of the two diseases show that the relative extents of factors considered in them are similar. Figure 7c further shows the paired connections between climate and non‐climate abiotic factors involved in the studies. Land factors (45%) are considered along with climate factors in many studies on LD, but water and socioeconomic factors are rarely included (14% and 6% respectively). For cryptosporidiosis, water factors (42%) and socioeconomic factors (29%) are relatively often considered along with climate factors, while land factors are largely omitted (13%).

**Figure 8:**
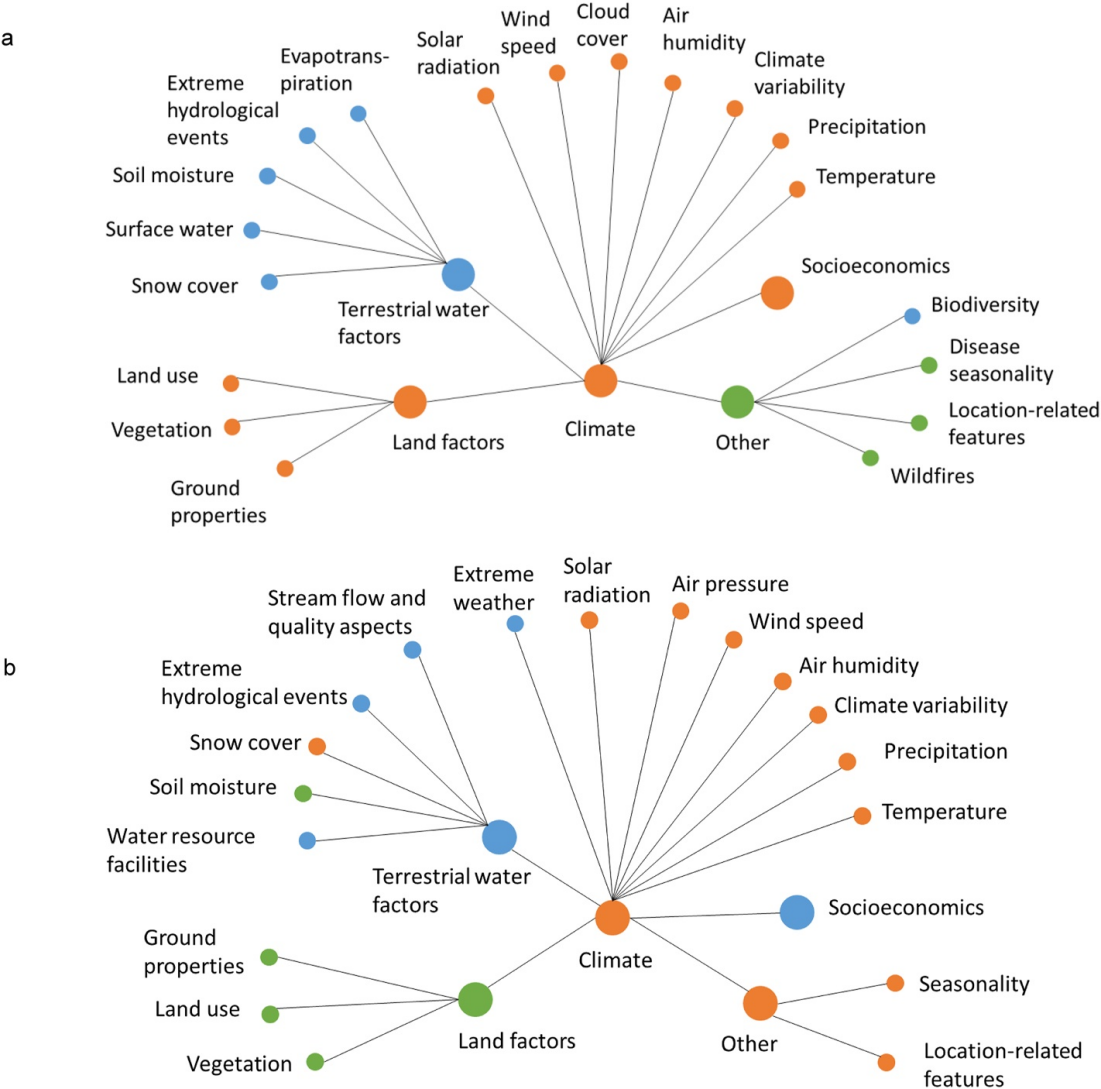
Network diagrams of factors (larger nodes) and variables (smaller nodes) considered in studies of (a) Lyme disease and (b) cryptosporidiosis, following the factor/variable structure in Table 1. Nodes with the same color emerge belonging to and being connected within the same factor/variable community.

The network diagrams in Figure 8 further show the linkages between different factors and variables considered in the studies. For LD (Figure 8a), terrestrial water factors are not well integrated with climate factors; the water factors emerge as a different factor/variable community (blue), related more to biodiversity than climate (orange). In contrast, land and socioeconomic factors emerge as rather well integrated with climate factors for LD.

**Figure 9:**
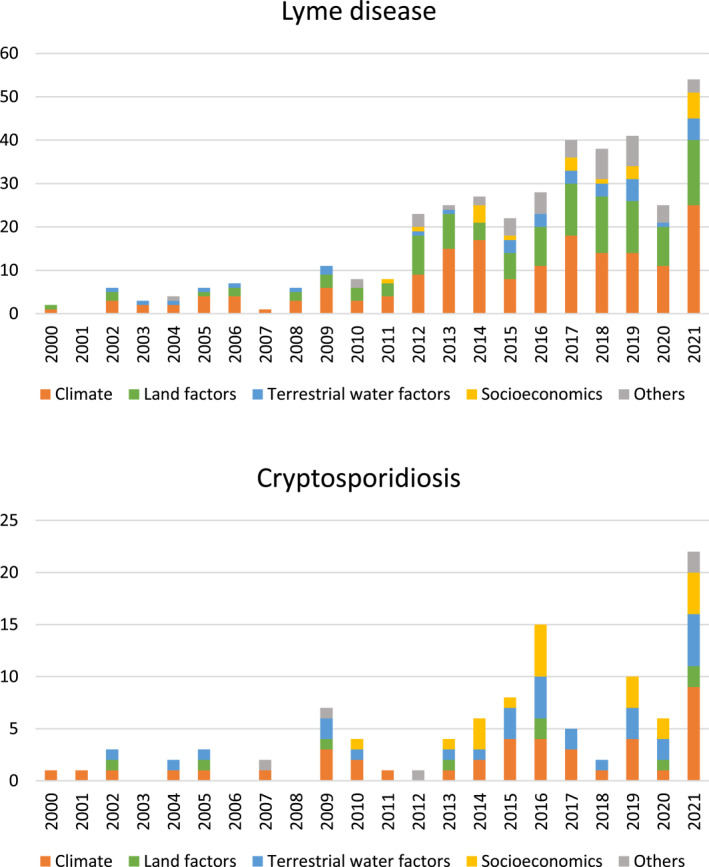
Distinction of different factors included in quantitative studies over time for (upper panel) Lyme disease and (lower panel) cryptosporidiosis.

For cryptosporidiosis (Figure 8b) there is a different pattern of climate integration with other environmental and anthropogenic driver‐pressure factors. In consistency with the chord diagram results, climate factors are more connected with water factors in these studies, in particular through the water variable of snow cover (orange as the climate community) and the climate variable of extreme weather (blue as the water community), and also linked more to socioeconomic factors for cryptosporidiosis than for LD, while land factors emerge as a separate factor/variable community (green). Overall, the disease studies reviewed here are climate‐centered but address also, to some degree, disease sensitivity to other relevant environmental and anthropogenic driver and pressure factors. Our quantification of understudied and/or unconnected relevant other factors points at important research gaps that need to be addressed in further research as insufficient knowledge about them can lead to misinterpretations of infectious disease sensitivity to those as well as to climate factors. This need does indeed also appear to be increasingly realized by researchers, as there is clear trend of more recent studies increasingly integrating more relevant factors, even though their shares of the total published papers are still small (Figure 9).

### 3.4 Transmission Pathway Components

Understanding of the infectious disease impacts of climate change at the scale of whole communities of hosts and parasites is still in early development (Rohr et al., 2011), as also seen in our results, even though each component plays important role in the consequent risks.

**Figure 10:**
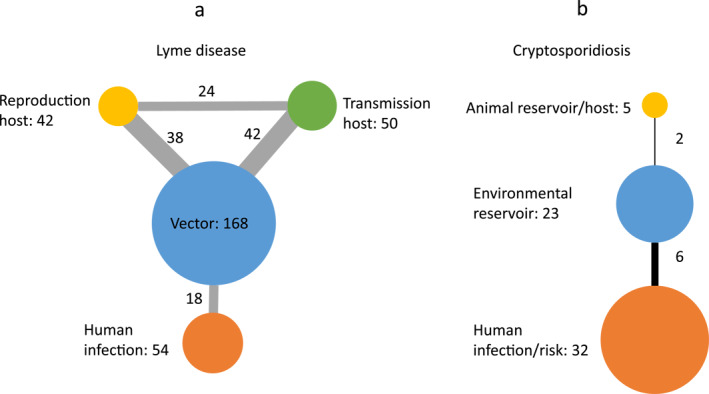
Counts of studies on transmission components and their interconnections for (a) Lyme disease and (b) cryptosporidiosis.

For LD, climate‐driven changes in abundances of ticks and host (Kilpatrick et al., 2014; Stafford et al., 1998), and in their distributions (Killilea et al., 2008; Kitron & Kazmierczak, 1997) and migration patterns (Hahn et al., 2016; Khatchikian et al., 2012; Madhav et al., 2004) will affect the risk of LD infections. In addition, species behavior and interactions can also change the risk. For example, *I*. *scapularis* in the United States infests highly on efficient LD transmission hosts (rodents and shrews) in the north but not in the south, causing decline in tick infection prevalence from north to south (Ginsberg et al., 2021). Furthermore, climate‐associated variability in the timing of *I*. *scapularis* host seeking also contributes to geographic heterogeneities in LD risk (Gatewood et al., 2009). Amplification or dilution effects caused by changes in host composition are also a big concern (Altizer et al., 2011; Bellard et al., 2012). Both amplification and dilution have been reported, with the outcome depending on competition mechanisms, host contact rates with ticks, and acquired host resistance to ticks (Ogden & Tsao, 2009; Wood & Lafferty, 2013). In our result, a majority of reviewed studies (168, 81% of the quantitative studies) address climate change effects on the vector, that is, ticks (Figure 10a). This is reasonable, since the distribution, reproduction and behavior of ticks are influenced by climate (Brownstein et al., 2005; Ostfeld & Brunner, 2015). However, reproduction hosts (e.g., white‐tailed deer) and transmission hosts (e.g., white‐footed mouse) are considerably less studied (42 and 50 studies, respectively). This is an important research gap, as infected hosts may play a great role in transmission as mentioned before, sometimes even more than ticks in the further expansion and naturalization of LD (Humphrey et al., 2010). There is also a lack of studies on the connections between vectors and human infections, with only 18 studies identified to consider both.

Cryptosporidium has been widely detected in livestock and wild animals (Ryan et al., 2014). Although many outbreaks have been either due to exposure to farm animals or contact with polluted water sources by livestock (Goh et al., 2004; Ong et al., 1999), wild animals also provide a reservoir of infection for domestic animals through contamination of food or shared environments (Mosier & Oberst, 2000). For example, a cryptosporidiosis outbreak in the UK raised awareness of the need to investigate rabbits as a transmission source (Chalmers et al., 2009). Since oocysts can survive in the environment for long time, cryptosporidiosis is also strongly associated with precipitation (Jagai et al., 2009; Naumova et al., 2005; Tryland et al., 2011). Figure 10b shows 32 studies (62% of the quantitative ones) consider human infection, 23 studies (44%) environmental reservoirs, and only 5 publications (10%) animal reservoirs. The connections between these transmission components are further rarely studied. The lack of studies on animal reservoirs is a notable research gap.

### 3.5 Geographic Study Distribution

The quantitative studies with focus on specific regions comprised in total 184 articles for LD and 42 for cryptosporidiosis.

**Figure 11:**
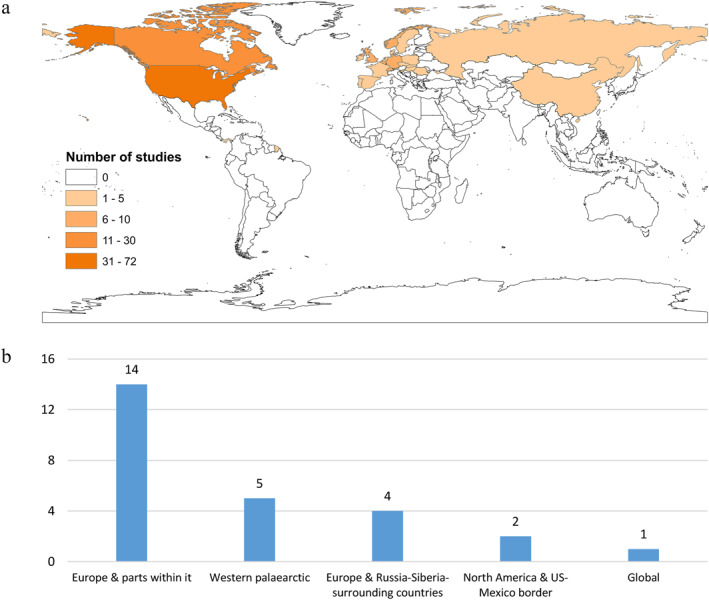
Number of studies on Lyme disease for (a) specific countries and (b) larger transboundary regions.

For LD, the studies are largely focused on the Northern Hemisphere (Figure 11), which corresponds to the main geographic distribution of the primary vector (Schotthoefer & Frost, 2015). Most studies (97%) focus on Europe (the most studied continent for this disease) and North America (Figure 11), with only four studies focusing on Asia (Estrada‐Peña et al., 2013, 2015; Yang et al., 2020, 2021). Lyme disease is a common disease in North America, with approximately 476,000 cases per year in the United States (Kugeler et al., 2021; Schwartz et al., 2021), while Europe has around 85,000 cases per year (Lindgren & Jaenson, 2006). Reports of LD are still relatively rare in Japan and Korea (Stone et al., 2017), while the disease is clearly established in China even though exact numbers have yet to be reported (Fang et al., 2015). Since LD is found throughout Europe, Russia and Asia in the Northern Hemisphere (Schotthoefer & Frost, 2015), the future risk in the less studied regions, such as in Asia, requires further attention.

**Figure 12:**
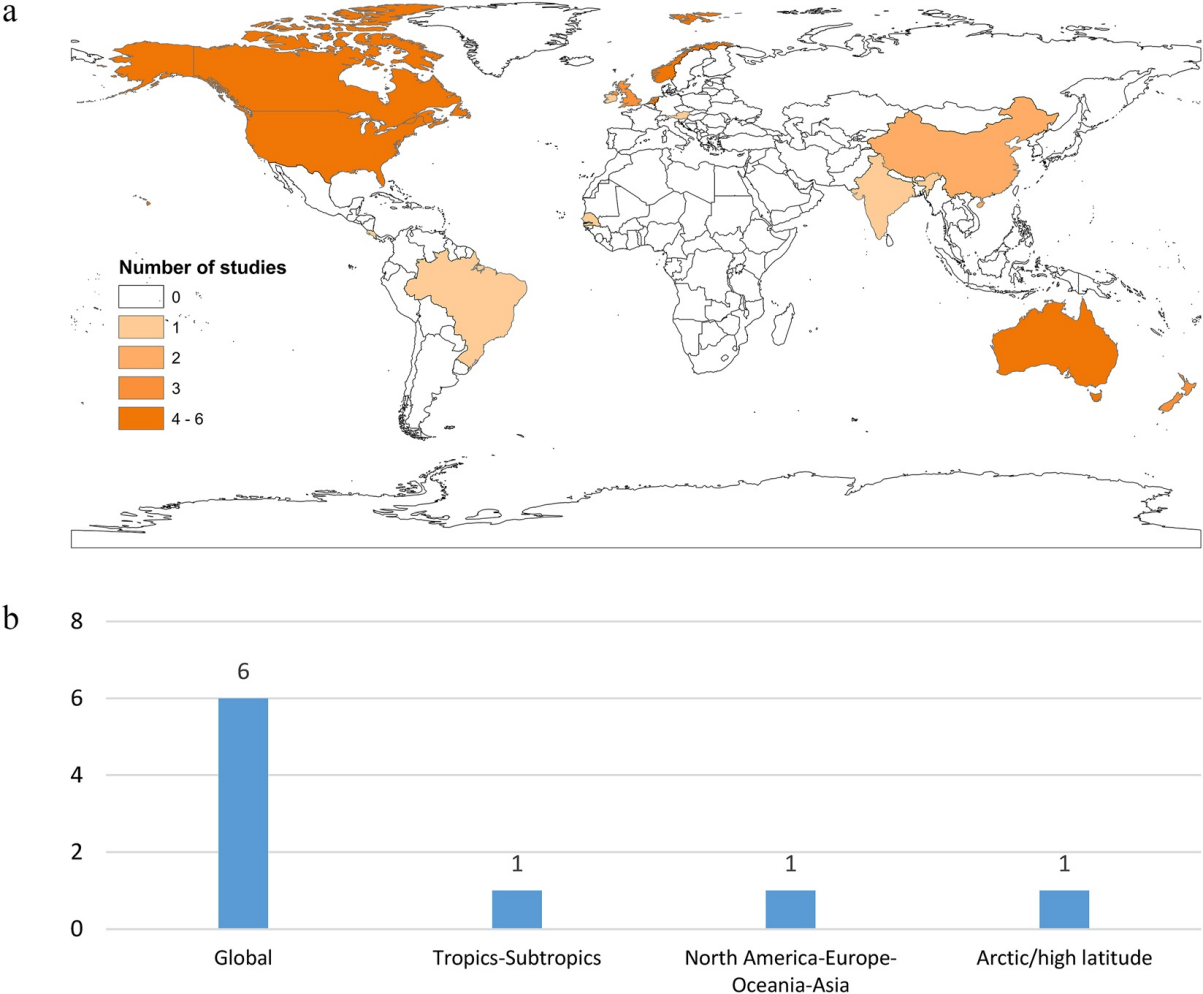
Number of studies on cryptosporidiosis for (a) specific countries and (b) larger transboundary regions.

For cryptosporidiosis, the studies emerging from the scoping review have a wider geographic distribution and are overall mostly large‐scale, including also relatively many global assessments (Figure 12). This is consistent with the wide geographic spreading of cryptosporidiosis across all continents except Antarctica (Fayer, 2004). A study of Global Burden of Diseases, Injuries, and Risk Factors (Khalil et al., 2018) uses disability‐adjusted life‐years as a metric for quantifying the global burden of cryptosporidiosis among children younger than 5 years, who are more vulnerable to the infection (Thompson et al., 2005). The study found large variability in disease burden between countries, with the highest burden in the Sahel region of sub‐Saharan Africa and in central sub‐Saharan Africa, even though cryptosporidiosis is globally distributed. In high‐income countries, cryptosporidium is not found to pose a major diarrhea burden (Khalil et al., 2018). However, studies focusing on Africa are largely lacking, even though cryptosporidium infection in children is high there compared with other continents (Khalil et al., 2018).

## 4 Discussion and Conclusions

This study has developed a scoping review approach to quantifying research effort and identifying key research gaps for the dependence and sensitivity of infectious diseases to climate and other driver‐pressure changes occurring around the world. For LD, there is a lack of studies considering terrestrial water and socioeconomic factors in addition to and combination with the climate factors. For cryptosporidiosis, there is a lack of studies considering land factors and their linkages with climate change. The climate and other driver‐pressure interactions with host and parasite communities are understudied for both diseases, as are also important regions in relation to the geographic disease distribution around the world. In particular, Asia and Africa emerge as main geographic research gaps for LD and cryptosporidiosis, respectively. Method‐wise, there are relatively few studies that provide new laboratory or field data, and quantitatively synthesize or meta‐analyze available models and data or use them for projection of future disease evolution under different scenarios of climate and other driver‐pressure changes.

With regard to understudied driver‐pressure factors, there are relatively few quantitative studies on water factors for LD, despite reported impacts of, for example, droughts and soil moisture on tick survival (Süss et al., 2008). For cryptosporidiosis, terrestrial water and climate factors have been more connected in the reviewed studies, but primarily just with regard to snow cover and extreme weather, while there is still disconnect between other water and climate variables. Socioeconomic factors are also rarely considered in studies of LD, even though they can play an important role in the disease prevalence. For example, LD incidences in the UK, US, and Canada are found to be associated with socioeconomic status and rural living conditions (Slatculescu et al., 2022; Springer & Johnson, 2018; Tulloch et al., 2019). Societal contributions to raising public awareness and learning in order to mitigate risks with appropriate behavior can also greatly contribute to effectively decreasing infection level (Sharareh et al., 2017). Projections of LD risk under future climate scenarios can also contribute to this end. Such contributions have shown higher future risks in the USA and Europe, for example, regarding higher incidences (Dumic & Severnini, 2018; Robinson et al., 2015), shifted seasonality (MacDonald et al., 2020; Monaghan et al., 2015), and expanded geographical distribution of vectors (Alkishe et al., 2017; Gardner et al., 2020; Hahn et al., 2021; Jaenson & Lindgren, 2011; MacDonald et al., 2020; Popov & Yasyukevich, 2014; Porretta et al., 2013). However, disease projections considering socioeconomic aspects reveal large uncertainties about future risks due to unknown and largely unpredictable uses of the human mitigation capacities (Couper et al., 2021; Li et al., 2019). For cryptosporidiosis, land factors emerge as less studied, even though they can be essential for controlling pathogen survival in the environment and along spreading pathways to water sources. For example, soil texture combined with temperature plays an important role in oocyst survival (Jenkins et al., 2002). Soil and vegetation cover can further act as filter for pathogen removal (Cilimburg et al., 2000) or prevention from entering drinking water sources. Alternatively, soil can protect the pathogen by trapping it within the soil column, which in turn implies an important connection with precipitation that can counteract the trapping by remobilizing the oocysts (King & Monis, 2007).

With regard to transmission pathways, studies on the hosts are lacking for both diseases. For LD, a northward vector expansion has been reported and projected for North America and Europe (Alkishe et al., 2017; Brownstein et al., 2005; Jaenson & Lindgren, 2011; Lindgren et al., 2000; Popov & Yasyukevich, 2014). However, the future evolution of, so far relatively understudied, host population density will ultimately determine whether the vector expansion persistence after introduction. Moreover, even if host population density can shift in the same direction as vectors, interactions within the local ecosystem need to be studied as they may have either a strengthening effect, where risk increases locally, or a dilution effect, where low‐reservoir competent wildlife dominates and the risk is reduced (LoGiudice et al., 2003). For cryptosporidiosis, there is a general scarcity of data on prevalence in wild animals, even though the sparse available data indicate this pathogen to be circulating in wildlife populations that can be essential contributors to environmental pools threatening humans (Salyer et al., 2012; Samra et al., 2011).

The identified Asian geographic gap of studies on LD may be due to relatively low disease prevalence, especially in East Asia (Stone et al., 2017), but the disease has been shown to be clearly established in China (Fang et al., 2015). Overall, evaluation of future LD risks in Asia is required because the general factors that drive and escalate prevalence of this disease are similar in Asia as in the current research hotspots of North America and Europe (Kulkarni et al., 2015; Mannelli et al., 2012; Medlock & Leach, 2015), for example, with large parts of Asia projected to experience similar climate warming as North America (Allan et al., 2021). The lack of studies on cryptosporidiosis in Africa is not due to low prevalence, but primarily due to limitations in technological capabilities and surveillance systems (Schaefer et al., 2020). Considering the generally high background prevalence of diarrheal disease in Africa, it might also be more difficult to specifically identify a cryptosporidiosis outbreak there unless it is extremely dramatic. In addition, not all affected individuals seek medical support and, even if doctors obtain stool samples, the samples may not be appropriately analyzed (Chalmers, 2012).

In general, lack of consideration of other relevant driver‐pressure changes, in addition to and combination with climate change, can lead to misinterpretation of climate‐sensitivity results and implications. For example, for cryptosporidiosis, oocyst survival may differ for the same temperature in different soil types (Jenkins et al., 2002), and reliable prediction of LD incidence requires also consideration of human interactions with fragmented forest landscapes (MacDonald et al., 2019). Even though integration of all possible driver‐pressure factors may be unrealistic and can also introduce more uncertainties, more research is needed for developing improved capability to consider, distinguish between and predict disease impacts of and sensitivity to climate and other relevant driver‐pressure changes. Prediction and mitigation of climate change impacts on diseases cannot focus solely on purely climatic considerations but must also account for the influences of other driver‐pressure changes, such as demographic shifts, civil unrest, and changes in land use and water use and availability (Gage et al., 2008).

In terms of mitigation, vaccine is an effective way to mitigate the risk of infection. Unfortunately, for both diseases, there is no vaccine available in the market yet (CDC, 2022; Innes et al., 2020). One vaccine for LD called VLA15, made by Pfizer and Valneva, is still undergoing clinical trials (CDC, 2022). The vaccine development for cryptosporidiosis is even more challenging because protective immune responses to this parasite are incompletely understood so far, and this understanding needs to improve for efficient vaccine development (Mead, 2010; Pantenburg et al., 2008).

Overall, research on LD and cryptosporidiosis faces three common major challenges with the whole field of climate‐disease interactions. The first is the scarcity of long‐term time series of relevant data (Rodó et al., 2013). Disease surveillance systems differ between countries (Blanchard et al., 2022). Even in high‐income countries, for instance, in the US, LD became a national notifiable disease first in 1991 (Schwartz et al., 2017) and cryptosporidiosis became that even later, in 1995 (Dietz & Roberts, 2000). Considering seasonal‐to‐decadal climate cycles (Reason et al., 2006) and climate sensitivity of species dynamics (Visser, 2016), the now available short‐term disease records since those times are still insufficient for capturing the disease response to long‐term climate change trends. Human genome variations, which do not influence infections, may still affect infection severity and, as such, lead to surveillance inaccuracies that also need to be noted (Oosting et al., 2016; Wojcik et al., 2020).

The second major challenge is the complexity of integrating relevant theoretical, observational and experimental approaches for prediction of disease risk changes. Finally, the third challenge is the combined uncertainties associated with the whole chain of different involved driver, pressure and impact factors. To guide further climate‐disease research towards meeting these challenges, this scoping review has identified some important gaps in climate and other driver‐pressure change considerations and connections, along with gaps in the disease transmission components and geographies, with focus on LD and cryptosporidiosis. Bridging such gaps will enhance data availability to more relevant and comprehensive data availability, improve our capability for mechanistic data interpretation and predictive modeling, and enable us to “zoom out” and obtain a fuller picture of the whole complex climate‐disease interaction system.

## Conflict of Interest

The authors declare no conflicts of interest relevant to this study.

## Data Availability

The data for this scoping review were obtained and are available from performing the same searches in Web of Science™ (WoS) for publications from 1 January 2000 to 10 February 2022, with search terms: ((“borreliosis” OR “Lyme disease”) AND (“climate” OR “climate change” OR “climate variability”)) for LD, and ((“cryptosporidiosis” OR “cryptosporidium” OR “crypto.”) AND (“climate” OR “climate change” OR “climate variability”)) for cryptosporidiosis. The search yielded 555 publication results for LD and 185 for cryptosporidiosis that were further categorized as outlined and with category numbers stated and shown in the main text. The relevant reference data for and information extracted from these publications for the analysis in this study are available in an open access database (Ma et al., 2022 [database]; https://doi.org/10.5281/zenodo.7875759). Based on those category number results, bar charts in Figures 3, 4, 5, 6, 9, 11, and 12 were made with Microsoft Excel 2016. Figures 1, 2, 6, 8, and 10 were created or edited by Microsoft PowerPoint 2016. Chord diagrams in Figure 7 were created using the HoloViews Python library, post‐processed in Inkscape. Maps in Figures 11 and 12 were made with ArcMap 10.5.1 (https://www.esri.com/en-us/home). The Louvain algorithm was run through Gephi (https://gephi.org/) to provide results for creating Figure 8.
